# Enantiomeric Discrimination in Insects: The Role of OBPs and ORs

**DOI:** 10.3390/insects13040368

**Published:** 2022-04-08

**Authors:** Cassie Sims, Michael A. Birkett, David M. Withall

**Affiliations:** 1 Biointeractions and Crop Protection Department, Rothamsted Research, Harpenden, Hertfordshire AL5 2JQ, UK; cassie.sims@biol.lu.se (C.S.); mike.birkett@rothamsted.ac.uk (M.A.B.); 2 School of Chemistry, University of Nottingham, University Park, Nottingham NG7 2RD, UK

**Keywords:** insect, olfaction, chemosensory, odorant receptors, odorant-binding proteins, chiral, enantiomeric discrimination, chemical ecology

## Abstract

**Simple Summary:**

Insects use olfaction, i.e., their sense of smell, to detect odors that elicit behavioral responses, with structurally similar compounds eliciting different responses. The roles of specific recognition proteins, i.e., odorant-binding proteins (OBPs) and odorant receptors (ORs), located in insect antennae, in discriminating between structurally similar compounds are not fully understood. Here, we explore current research in understanding the role of OBPs and ORs in discriminating between enantiomers—mirror image structures—in insect chemical ecology and chemoperception.

**Abstract:**

Olfaction is a complex recognition process that is critical for chemical communication in insects. Though some insect species are capable of discrimination between compounds that are structurally similar, little is understood about how this high level of discrimination arises. Some insects rely on discriminating between enantiomers of a compound, demonstrating an ability for highly selective recognition. The role of two major peripheral olfactory proteins in insect olfaction, i.e., odorant-binding proteins (OBPs) and odorant receptors (ORs) has been extensively studied. OBPs and ORs have variable discrimination capabilities, with some found to display highly specialized binding capability, whilst others exhibit promiscuous binding activity. A deeper understanding of how odorant-protein interactions induce a response in an insect relies on further analysis such as structural studies. In this review, we explore the potential role of OBPs and ORs in highly specific recognition, specifically enantiomeric discrimination. We summarize the state of research into OBP and OR function and focus on reported examples in the literature of clear enantiomeric discrimination by these proteins.

## 1. Introduction

Insects use chemistry to communicate in sexual reproduction, prey and natural enemy location/avoidance, oviposition and host location. Insect chemical communication occurs mainly via olfaction, i.e., the recognition and discrimination of olfactory ligands by olfactory proteins mainly located in the antennae. Although insects possess other chemosensory systems, such as the gustatory (taste) system, olfaction remains one of the most critical senses in insects [1,2]. Elucidating the chemistry and physiology underpinning insect olfaction provides an opportunity to better understand insect behavior [3]. Furthermore, as many insect species are either agricultural pests or harmful to human health, understanding insect olfaction also provides an opportunity to develop novel pest management approaches that are effective, economically viable and sustainable [4].

Chirality is common in nature and many insect species utilize chiral olfactory ligands for communication [5,6]. Furthermore, in contrast to hormones or other internal chemical communication pathways, insect olfaction is an external recognition process, meaning that olfactory ligands need to be recognized amongst a plethora of background signals. This implies that the olfactory recognition system in insects involving olfactory proteins must be highly sophisticated [5].

Though enantiomeric discrimination is a critical biochemical process, the mechanisms by which olfactory proteins can discriminate between structurally similar compounds with high levels of specificity remain unclear. The complex, membrane-bound nature of some olfactory proteins provides a challenge for their study in vitro [7,8], meaning that very few membrane-bound protein structures have been reported to date [7,9]. Furthermore, although extensive studies have been conducted on soluble olfactory proteins and their interaction with ligands [10,11,12,13,14,15], the specific role of such proteins remains largely debatable, with some proteins demonstrating high levels of specificity and others being highly promiscuous [16,17,18]. Where extensive functional analysis of olfactory proteins has been conducted, studies have frequently neglected to screen multiple stereoisomers or enantiomers of an olfactory ligand, most likely due to the expense and difficulty of synthesizing non-naturally occurring enantiomers, compared to naturally-occurring enantiomers which are often easily derived from plant sources [19].

Understanding how enantiomeric discrimination arises, specifically the role of membrane-bound and soluble olfactory proteins, will provide a greater fundamental understanding of how sophisticated insect olfactory recognition systems function. This could lead to novel practical applications, such as monitoring and management of significant agricultural pests [4].

## 2. Chirality in Insect Olfaction

### 2.1. Semiochemistry

Chemical communication between organisms is mediated by semiochemicals that can be categorized into either pheromones, which are compounds that are released by an organism and induce a response in individuals of the same species, or allelochemicals, which induce responses in individuals of different species [20,21]. Different types of pheromones include sex pheromones, aggregation pheromones and alarm pheromones [20]. Pheromones can be further categorized into releasers, which induce an immediate behavioral change, and primers, which initiate a complex set of physiological or developmental changes but may result in no immediate behavioral change [21]. Allelochemicals can also be further categorized depending on whether the emitter or receiver is the beneficiary [21]. Semiochemicals are used in sexual reproduction, prey and natural enemy avoidance, oviposition and host location. Though widely employed by insects for communication, semiochemicals have been identified in many other organisms including mammals, birds and fish [22,23,24]. Many semiochemical-based interactions, especially pheromone interactions, are highly specific. This specificity may arise from the use of species-specific compounds, or the use of the same compounds but at either different ratios/concentrations or with different stereochemistries [6,25].

### 2.2. Chiral Pheromones

**Figure 1:**
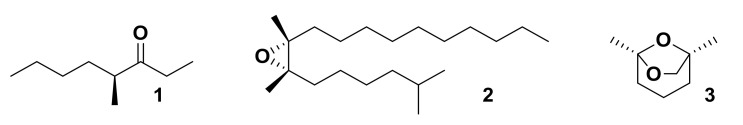
Early synthesized enantiomerically pure pheromones, *Atta texana* alarm pheromone (*S*)-4-methyl-3-heptanone **1**, *Lymantria dispar* sex pheromone (7*R*,8*S*)-(+)-disparlure **2** and *Dendroctonus brevicomis* aggregation pheromone (1*S*,5*R*)-frontalin **3** .

Insect species across a broad range of taxa utilize chiral pheromone components. Identification of the specific stereochemistry and bioactivity of chiral pheromones requires the synthesis of specific enantiomers of pheromone components [6]. Synthesis of enantiomerically pure pheromones was first achieved in the 1970s with the synthesis of compounds such as (*S*)-4-methyl-3-heptanone **1**, the alarm pheromone of leaf-cutting ant *Atta texana*, (7*R*,8*S*)-(+)-disparlure **2**, the sex pheromone of the female gypsy moth *Lymantria dispar* and (1*S*,5*R*)-frontalin **3**, an aggregation pheromone of the western pine beetle, *Dendroctonus brevicomis* (Figure 1) [6,26,27,28]. Since then, hundreds of enantiomerically pure pheromone components have been synthesized [6].

**Figure 2:**
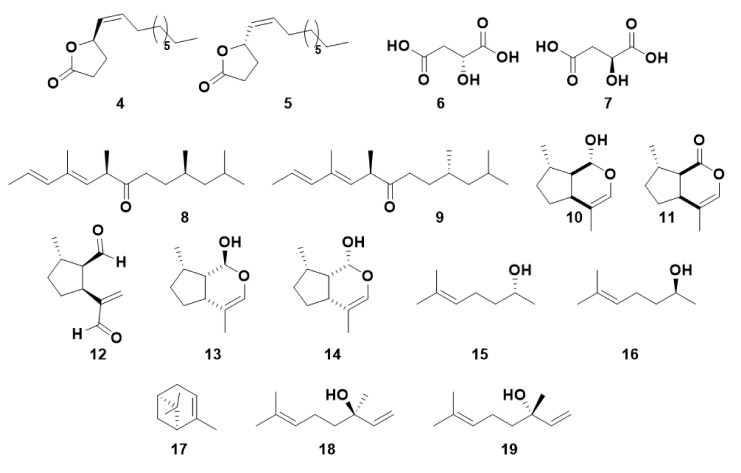
The chemical structures of a selection of chiral insect pheromones and semiochemicals involved in insect olfaction.(*R*)-Japonilure **4,** (*S*)-japonilure **5,** (*R*)-malic acid **6**, (*S*)-malic acid **7**, (6*R*,10*R*)-matsuone **8,** (6*R*,10*S*)-matsuone **9**, (1*R*,4a*S*,7*S*,7a*R*)-nepetalactol **10**, (4a*S*,7*S*,7a*R*)-nepetalactone **11**, (1*R*,2*S*,*5S*)-dolichodial **12**, (1*S*,4a*R*,7*S*,7a*S*)-nepetalactol **13** and (1*R*,4a*R*,7*S*,7a*S*)-nepetalactol **14**, (*R*)-sulcatol **15**, (*S*)-sulcatol **16**, α-pinene **17**, (*R*)-linalool **18** and (*S*)-linalool **19**.

Generally, insects utilize a specific enantiomer as a chiral pheromone component and do not respond to the other enantiomer in the same way [6]. However, the other enantiomer may inhibit pheromone activity, may have differing effects on males and females, or have variable levels of bioactivity dependent on their stereochemical similarity to the most active enantiomer [6]. This level of variation of bioactivity of pheromones clearly indicates a complex recognition system, the mechanism and specificity of which may vary between different species. The attractiveness to males of (*R*)-japonilure **4**, the female-produced sex pheromone of the Japanese beetle, *Popillia japonic*a, is inhibited by (*S*)-japonilure **5**, resulting in a lack of biological response when a racemate is used to study beetle behavior [29] (Figure 2). Even minor contamination by (*S*)-japonilure can inhibit male responses to (*R*)-japonilure. The olive fruit fly, *Bactrocera oleae*, produces a racemic mixture of (*R*) and (*S*)-malic acid (**6** and **7)**, with the (*R*)-isomer acting as the male-attractive sex pheromone and the (*S*)-isomer attracting females [30]. (6*R*,10*R*)-Matsuone **8** is a sex pheromone of red pine scale, *Matsucoccus resinosae*, whereas the (6*R*,10*S*) isomer **9** is active but to a lesser degree [31].

Pheromones may also comprise mixtures of different compounds or mixtures of enantiomers. Aphids utilize a sex pheromone, generally consisting of two components (1*R*,4a*S*,7*S*,7a*R*)-nepetalactol **10** and (4a*S*,7*S*,7a*R*)-nepetalactone **11** [32,33]. Some aphids, such as the rosy apple aphid, *Dysaphis plantaginea* and the damson-hop aphid, *Phorodon humuli*, also employ other isomers including (1*R*,2*S*,*5S*)-dolichodial **12**, (1*S*,4a*R*,7*S*,7a*S*)-nepetalactol **13** and (1*R*,4a*R*,7*S*,7a*S*)-nepetalactol **14** [25,34]. Although the aphid sex pheromone components are ubiquitous across most species, the ratio of the components is species-specific [19,35]. For example, for the pea aphid, *Acyrthosiphon pisum*, the sex pheromone consists of a 1:1 ratio, whereas the black-bean aphid, *Aphis fabae*, uses a very high ratio of lactone to lactol [35]. Though some pheromones comprise two different compounds with the same stereochemistry in all shared positions, some pheromones may comprise a mixture where both enantiomers are necessary for a biological response, eg. Sulcatol, the aggregation pheromone of the ambrosia beetle, *Gnathotrichus sulcatus*, comprises a 35:65 mixture of (*R*)-sulcatol **15** and (*S*)-sulcatol **16** respectively [36].

In addition to volatile pheromone components, some insect species, mainly Hymenoptera, utilize cuticular hydrocarbons (CHCs) for intraspecific communication. Stereochemistry is often conserved within a species, eg. in a study of 36 methyl-branched CHCs across 20 species, all CHCs were found to possess (*R*) stereochemistry [37].

### 2.3. Other Chiral Semiochemicals

Chirality is also crucial for the bioactivity of other semiochemicals eg. plant volatile organic compounds (VOCs), which play a significant role in plant-insect interactions. Insect host location required for pollination, herbivory or oviposition may require high levels of specificity in semiochemicals similar to that for intraspecific interactions [38,39]. Dependent on the plant-insect relationship, some plant VOCs may be attractive and some may be repellent to insects [38].

Plant VOCs can be identical to pheromone components, such as (4a*S*,7*S*,7a*R*)-nepetalactone **11** which, in addition to its role as an aphid sex pheromone component, is emitted by the catmint plant *Nepeta cataria* and repellent to many insect species [40,41]. One stereoisomer of α-pinene **17**, a plant-produced isoprenoid, is also known as an aggregation pheromone component for some *Ips* species of bark beetle [42]. Furthermore, other Coleoptera species, such as the red turpentine beetle, *Dendroctonus valens*, show differential responses to enantiomers of α-pinene [43]. Additional VOCs of isoprenoid origin may have differing effects depending on their chirality. Linalool, which can exist as either the (*R*)-**18** or (*S*)-**19** form, has been frequently shown to have different behavioral or physiological activities depending on which enantiomer is utilized [44,45].

## 3. Insect Olfactory Proteins

### 3.1. Introduction to Insect Olfaction

Olfaction (i.e., the recognition and discrimination of olfactory ligands) is critical for the perception of pheromones and other semiochemicals. Prior to the identification of the first insect pheromones, little was understood regarding the mechanisms underlying insect olfaction, with knowledge being mainly based on anecdotal evidence [46].

**Figure 3:**
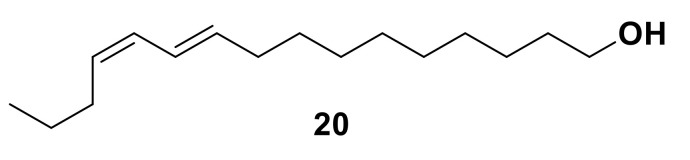
The chemical structure of Bombykol **20**, the first insect pheromone isolated from the silkworm moth, *Bombyx mori*.

The first insect pheromone to be characterized was Bombykol ((10*E*,12*Z*)-hexadec-10,12-dien-1-ol) **20** (Figure 3), identified from the silkworm moth, *Bombyx mori* [15,46,47,48]. Due to the large physical size of this insect, a significant quantity of pheromone was isolated and initial structural identification was achieved using infrared (IR) and ultraviolet (UV) spectroscopy. Since the first identification of pheromones, significant advances in the field of olfaction research were made by developments in analytical chemistry. Introduction of techniques for the identification of complex chemical structures, particularly nuclear magnetic resonance (NMR) spectroscopy and coupled gas chromatography-mass spectrometry (GC-MS) for the detection of minute quantities of compounds, were used to confirm the identification of Bombykol **20** [15,46,47], and vastly expanded the known chemical library of pheromones and other semiochemicals [46]. The identification of the previously discussed aphid sex pheromone components, (1*R*,4a*S*,7*S,*7a*R*)-nepetalactol **10** and (4a*S*,7*S*,7a*R*)-nepetalactone **11**, relied on such techniques [32,49].

The main olfactory organs in insects are the antennae, although some insects possess additional olfactory organs, such as maxillary palps [50,51]. Insect antennae generally comprise segments and are covered in small hair-like structures called sensilla [17,51,52,53,54]. Olfactory sensilla are perforated with pores, through which odorant molecules diffuse. Each sensillum can be uniporous (one pore) or multiporous (many pores) [17,52]. Sensilla contain an aqueous fluid, or sensillum lymph, in which olfactory receptor neurons (ORNs) are bathed, and where olfactory proteins (i.e., odorant-binding proteins (OBPs) and olfactory receptors (ORs)) are located [17]. There is no similarity between the OBPs and ORs in structure, but both appear to play a role in olfaction and interact with ligands, including pheromones and semiochemicals. Specific ORs have been mapped to specific sensilla types [17,51].

Diversity in function has been observed across sensilla. There are a few morphological classes of sensilla found in most insects; basionic, trichoid, placoid and coeloconic [17,50,51,54]. Each morphological class is generally responsible for a different function. In *Drosophila*, the basionic sensilla are responsible for fruit odors, trichoid sensilla for pheromones, and coeloconic sensilla for organic acid and amine-based odors [17]. For vetch aphids, *Megoura viciae*, placoid sensilla bear pores indicating a chemosensory role [53]. Variation in sensilla abundance and morphology can be observed between different insects that have different chemosensory profiles [54].

Olfactory neuroanatomy is an extensive field of research that focuses on insect olfaction, including ORNs that express ORs [50,51,55,56]. In Mammalia, ORNs are bipolar, allowing dendrites to give rise to numerous specialized cilia and providing a large receptive surface for the binding of odors to ORs [56]. Removal of these specialized cilia in mammals removes associated olfactory responses [56]. ORs are activated and hypothesized to generate an action potential, which travels along an ORN that glomerulates and converges in the brain [51,56,57,58].

Significant developments in the study of odorant perception have been furthered by recent advances in insect genomics. With full genome sequences now available for many insect species, including model organisms such as *Drosophila melanogaster*, many genes for OBPs and ORs have been identified [59]. Furthermore, by using advanced molecular biology techniques, the function of these receptors and proteins can be more extensively studied [17,18,58,59]. Advances in molecular and structural biology techniques allow for more intensive study of the structure and function of olfactory proteins, via heterologous expression, structural characterization and knockout or knockdown studies with RNAi and CRISPR/Cas9 gene editing [60].

### 3.2. Odorant-Binding Proteins

OBPs are a unique group of olfactory proteins found in high abundance in the sensillum lymph, the aqueous fluid that can be found within the sensilla of the antennae [61]. These proteins are highly structurally conserved across insects and the high abundance of mRNA encoding for OBPs found in the antennae suggests they play an important role in olfaction [17].

The first OBP to be identified in invertebrates was from an extract of the large moth, *Antheraea polyphemus*, using a radioactively labeled pheromone in ligand-binding experiments [61]. Initially, OBPs could be categorized by their six conserved cysteines, which results in a similar 3D structure despite diverging amino acid sequences [61]. However, further research showed a greater diversity of 3D structure, and three distinct categories have now been defined; classic OBPs (possessing six highly conserved cysteines that form disulfide bridges), Plus-C OBPs (possessing eight conserved cysteines and one conserved proline) and atypical (possessing nine or ten conserved cysteines) [62]. The conserved cysteines and multiple disulfide bridges lead to the high thermal stability of these proteins. OBPs range in size from approximately 110 to 240 residues, usually resulting in proteins of 10–25 kDa in size [62]. In addition to OBPs, another family of proteins, known as chemosensory proteins or CSPs, have been described. CSPs show similar binding activity to OBPs, but no sequence similarities, and only share four conserved cysteines [63].

Though thermally stable, OBPs are flexible globular proteins that may occur in multiple conformations. Alternate conformations may arise as a result of conditions such as pH change or binding activity. Pheromone-binding proteins (PBPs) found in *B. mori* and other insect species show pH-dependent conformational changes [12,64,65,66,67,68,69]. This change is often associated with the binding and release of a ligand [64].

**Figure 4:**
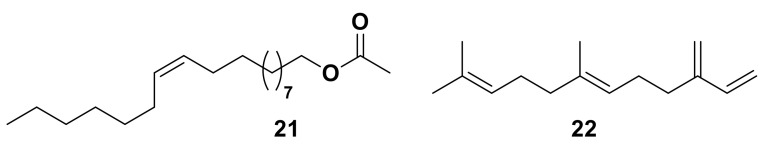
The chemical structure of (*Z*)-vaccenyl acetate (VA) **21**, the male sex pheromone of *D. melanogaster*, and (*E*)-β-farnesene **22**, the aphid alarm pheromone.

Numerous roles have been proposed for OBPs via studies on a range of insect species [16,18,61,70]. Many insect odorant molecules are highly lipophilic and are poorly soluble in aqueous solutions, which indicates these proteins may play a role in solubilizing or transporting the ligands to the odorant receptors in the aqueous sensillum lymph. OBPs have been shown to reversibly bind behaviorally active olfactory ligands, suggesting they play a role in olfactory perception [71,72,73,74,75,76,77]. LUSH, one of the most widely studied OBPs found in *D. melanogaster* [17,78,79], is thought to play a role in recognition and response to the male sex pheromone (*Z*)-vaccenyl acetate (VA) **21**, and has been shown to bind to VA *in vitro*, as well as other insect pheromones, short-chain alcohols and phthalates (Figure 4) [17]. *Bombyx mori* OBPs and PBPs are capable of discriminating between *B. mori* sex pheromone components and bombykol [74,80,81], whilst OBPs in aphids have been shown to discriminate between the alarm pheromone, (*E*)-β-farnesene **22**, and other ligands (Figure 4) [11,72,82,83]. Though OBPs have been shown to discriminate some ligands from others in binding assays, the specificity of OBPs varies widely and the fluorescence techniques utilized for studying OBP-ligand interactions are not optimal for observing subtle differences in ligand binding.

Expression of the gene encoding for a specific OBP can be suppressed, resulting in lower levels of gene expression, or the DNA encoding for the gene can be knocked out, entirely removing the expression of the gene [60]. Deletion may lead to reductions in electrophysiological responses, spontaneous neuron firing and behavioral effects [50,55,82,84,85,86,87]. However, some studies have shown olfactory neuron responses are still functional after deletion of LUSH, and LUSH-deficient mutants do not show any behavioral defects [17,55]. The existence of such contradicting studies only furthers the need for a thorough understanding of the role of OBPs.

In addition to suggesting roles for OBPs and CSPs in olfaction, genomic and proteomic studies generally show that many OBPs or CSPs are not found within the primary sensory organs (antennae) and are expressed in a wide diversity of spatial patterns, suggesting they possess roles beyond olfaction. For the honeybee, *Apis mellifera*, only 12 of 21 identified OBPs and two of six CSPs have been identified within the antennae [17,18,88]. Though some OBPs are expressed in the gustatory (taste) system of some species, as well as in larval chemosensory organs (*Drosophilia*), many OBPs and CSPs may possess entirely non-chemosensory roles [16,17,70,89]. OBPs and PBPs have been found in the pheromonal or ejaculatory glands of insects [70,90,91,92]. CSPs have also been linked to development [70,93]. In addition to pheromone transport and development, OBPs and CSPs play a role in a variety of other biological processes. These include anti-inflammatory action in disease-carrying insects, humidity sensing in *Drosophila* and other roles in nutrition, vision, migration and insecticide resistance [16,70,89].

### 3.3. Odorant Receptors

**Figure 5:**
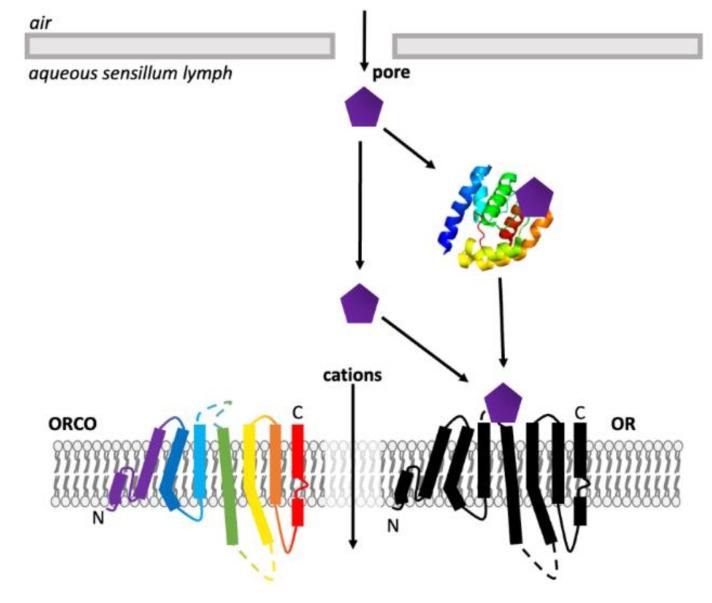
General olfactory processing in insects. Within the antennal sensilla, odorant-binding proteins (OBPs) play a role in allowing odorants to activate odorant receptors (ORs) which are co- expressed with the olfactory receptor co-receptor (ORCO). Once activated, an action potential travels along the odorant receptor neuron (ORN) to the antennal lobe.

ORs are found across the animal kingdom. For mammals, ORs belong to a group of proteins known as G-coupled protein receptors (GPCRs) [94], a large, diverse family of seven transmembrane (7TM) or heptahelical proteins with an extracellular N-terminus and intracellular C-terminus [56,95]. Although insect ORs are also 7TM proteins, they are distinct from GPCRs and possess an inverse heptahelical topology, with the N-terminus being located in the intracellular section of the transmembrane protein and the C-terminus found extracellularly (Figure 5) [7,9,58]. This suggests that insect ORs are a unique protein family, different from all other chemosensory receptors [58]. Insect ORs also differ in signal transduction and form a unique class of heteromeric cation channels [7,8,9,96].

ORs are a much larger and more diverse group of receptors in mammals. However, most ORs found in insects are co-expressed with another OR, known as ORCO (odorant receptor co-receptor, identified as OR83b in *D. melanogaster*) [1,58,97]. ORCO is structurally similar to other insect ORs, however, it is highly conserved across different insect species—only 20% conservation is seen between ORs, however approximately 60% shared identity can be seen for ORCO [7]. Basal insects lack an ORCO homolog due to their small number of OR genes, expressing homomeric OR complexes instead [9,98]. There is also no known mammalian ortholog of ORCO [58].

As with OBPs, knockdown or knockout studies contribute to the understanding of the function of ORs in insects. ORCO-deficient mutants show significant loss of olfactory function and related behaviors, indicating that ORCO must play an important role in olfactory signal transduction [99,100,101]. Antennal lobe glomeruli were also reduced in some ORCO-deficient mutants and antennal lobe size was affected by the knock-out of a sex pheromone receptor in *Spodoptera littoralis* [99,100,101,102]. OR binding activity is generally investigated using electrophysiological techniques, where the receptor is expressed in a membrane, exposed to a ligand and electrophysiological responses measured. Presently, a lack of structural data reduces options for in silico studies due to challenges associated with studying membrane-bound proteins [7,9,103,104,105].

## 4. Enantiomeric Discrimination by OBPs

OBPs are the first recognition proteins involved in the process of insect olfaction. After entering the antennae from the air via a pore, semiochemicals come into contact with OBPs contained within the aqueous sensillum lymph [18,61,70,106]. Although the specific role of OBPs in olfaction is unclear, they are capable of discrimination between different compounds and should therefore be evaluated for enantioselectivity [71,72,73,74,75,76,77]. OBPs exist in both insects and mammals, with similar structures. Mammalian PBPs have been shown to possess chiral discrimination properties, with a single residue conferring specificity to chiral compounds in a pig OBP [107]. However, OBPs are notoriously promiscuous binders, and finding an OBP capable of high levels of discrimination between any compounds is challenging.

### 4.1. OBP Specificity

Insects are capable of discriminating, at the behavioral and physiological level, between structurally similar compounds, including those with only small modifications or stereochemical differences, such as the sex pheromone components of aphids [32]. OBPs may assist in this process or potentially bind as an OBP-ligand complex to ORs. Supporting evidence for this hypothesis would include the occurrence of a conformational change, induced in OBPs when biologically active ligands bind. *D. melanogaster* OBP LUSH possesses a salt bridge between Lys87 and Asp118 that is only present in the apo (unbound) structure (non-VA-bound 3D- structure). When this salt bridge was disrupted in LUSH mutants, the DmOR69d neurons were activated in the absence of VA [55]. This suggests that LUSH is conformationally activated by VA, and in turn activates ORNs, or that a VA/LUSH complex interacts with ORNs [55]. OBPs and PBPs from different insect species also possess comparable salt bridges in their OBPs. *B. mori* possesses a salt bridge between Lys89 and Glu125 that is structurally analogous to LUSH [74].

Additional evidence for a conformation change, or conformation activation of OBPs generally indicates a C-terminal folding domain [13,64,74,108]. This C-terminus folding may be dictated by pH changes, where the acidic-residue-rich C-terminus loses negative charge at a low pH and forms an additional α-helix [108]. This additional helix can then enter the binding pocket and displace any ligand present [108]. This conformation activation has been observed in a range of species and could be responsible for interactions at the OR, where the OBP may expel the ligand for OR-binding, or the OBP may bind itself in a protein-protein interaction [66,67,68,69]. Some OBPs have also been shown to dimerize, and it is possible this dimerization could be disrupted by conformational changes, suggesting alternative ligand binding and release mechanisms [12].

OBPs generally have large binding pockets and can flexibly adapt to fit a multitude of ligands, often multiple ligands at once, leading to low levels of specificity [63]. It has been proposed that specificity might arise from numerous factors including the conformational changes resulting in active and inactive conformers, in addition to the energy minima and entropic contributions of ligands fitting into the ligand-binding pocket [109]. Ligands with lower conformational constraint, such as long-chain poly-unsaturated fatty acids that are often found as Lepidopteran pheromones, may be more difficult to discriminate compared with more conformationally constrained compounds, such as bicyclic terpenes. The more flexible ligands have more degrees of freedom, allowing them to fit into the available space within the ligand-binding sites. An OBP demonstrating high levels of discrimination will likely have a smaller binding site capable of accepting very specific, highly conformationally constrained compounds.

### 4.2. Enantiomeric Discrimination by Insect OBPs

**Figure 6:**
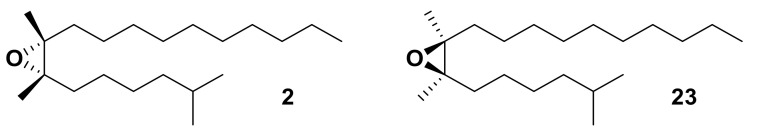
The chemical structures of (7*R*,8*S)-*(+)-disparlure **2** and *(*7*S*,8*R)-*(−)-disparlure **23**.

OBPs are not thought to be good candidates for enantiomeric discrimination [110]. Olfactory responses to different enantiomers occur within the same sensilla; two species of a scarab beetle, i.e., the Osaka beetle, *Anomala osakana* and the Japanese beetle, *Popillia japonica*, utilize different enantiomers of japonilure **4** and **5**, yet possess identical OBPs in the activated sensilla [110]. However, some OBPs and PBPs, have been identified that exhibit some degree of enantiomeric discrimination ability. PBP1 and PBP2, from the gypsy moth, *Lymantria dispar,* show differential responses to the sex pheromone component (*7R,8S)-*(+)-disparlure **2**, and its behaviorally antagonist enantiomer (*7S,8R)-*(−)-disparlure **23** (Figure 6) [10,111]. In a radioactive ligand-binding assay, LdisPBP1 was shown to have a higher affinity for (*7S,8R)-*(−)-disparlure **23** whereas LdisPBP2 responded with a higher affinity to (*7R,8S)-*(+)-disparlure **2**. Additionally, Apol-3, the PBP from the wild silk moth, *Antheraea polyphemus*, bound to both enantiomers with a low affinity, capable of discriminating both enantiomers [111]. Structural studies confirmed this binding activity and the conformational changes that these PBPs undergo at different pH, although LdisPBP1 was seen to bind both enantiomers of disparlure in NMR experiments, it bound the (-)-enantiomer with a higher affinity and interacts with different residues [10,109].

In the case of LdisPBPs and other examples, the kinetic activity of the protein, specifically the binding and release mechanism, may be important in determining specificity [10,12,67,108,112,113]. Furthermore, as the two enantiomers of disparlure result in different behavioral responses in gypsy moths, this would indicate that PBPs not only play a role in enantiomeric discrimination, but also suggests a discriminatory or recognition role in insect olfaction in general. Results thus far suggest that OBPs, in general, are not capable of enantiomeric discrimination, but there may exist PBPs with a high level of specificity. How the discriminatory ability of these peripheral proteins may affect olfactory responses in the insect is also unclear.

## 5. Enantiomeric Discrimination by ORs

Insect ORs play a significant role in determining insect responses to olfactory ligands. However, limited evidence is available confirming the structure of insect ORs, the specific activation of insect ORs by olfactory ligands and enantioselectivity in insect ORs. Enantioselectivity has been demonstrated in mammalian ORs, however, insect and mammalian ORs are vastly different in their structure and function [9,56,58,114,115]. Activation of ORs results in an electrophysiological response and, as it is clear that enantiomeric discrimination is critical in insect olfaction and unlikely to arise from OBPs, ORs are a better candidate for exploring enantioselectivity.

### 5.1. OR Specificity

**Figure 7:**
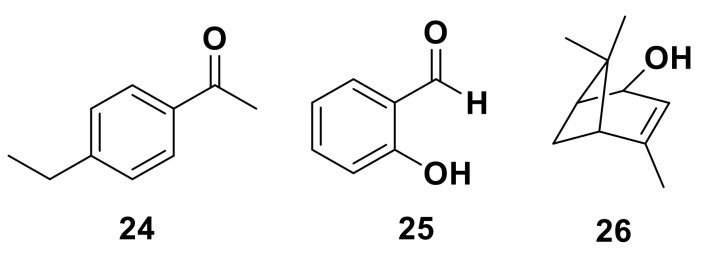
The chemical structure of 4-ethylacetophenone **24**, salicylaldehyde **25** and (*S*)-*cis*-verbenol **26**, compounds shown to elicit a response to the broadly tuned OR ApisOR4 from *A. pisum*.

Insect ORs can be broadly tuned or highly specific. Broadly tuned receptors show responses to a broad range of compounds, often with vastly different chemical structures [9,116,117]. For example, when ApisOR4, from the pea aphid, *A. pisum*, was screened against a panel of 57 odorants, it responded to a range of aromatic compounds, such as 4-ethylacetophenone **24** and salicylaldehyde **25**, in addition to bicyclic terpene compounds such as (S)-*cis*-verbenol **26** [118] (Figure 7). A total of six ligands were found to activate ApisOR4 with similar responses, all plant-derived volatiles [118]. Despite the binding of different ligands activating the same receptor, discrimination between ligands may come at a higher level. In mammals, odor perception has shown to be combinatorially coded (i.e., multiple ORs are activated in unique combinations as a response to a specific odor) [119]. Although little is known about odor coding in insects, evidence for distinctive neuronal perception between attractive and repellent odors has been identified [120].

By contrast, highly specific ORs respond to one or a few compounds, usually with a high level of specificity. This may include discrimination between similar chemistries, isomers or enantiomers. In Lepidoptera, there exist many ORs that are classified as pheromone receptors (PRs) due to their specific response to a single pheromone compound [117]. These receptors have been shown to discriminate between very similar chemical structures, though in Lepidoptera these are generally achiral compounds. Moth pheromones are generally Type I, straight-chain fatty alcohols and corresponding acetates and aldehydes, or Type II, long-chain polyunsaturated hydrocarbons, although there are exceptions (e.g., the sex pheromone (7*R,*8*S)-*(+)-disparlure **2** of the female gypsy moth *Lymantria dispar* and (1*S*,5*R*)-frontalin **3**). Highly specific receptors have conserved motifs, e.g., moth PRs show a conserved C-terminal region, the functional significance of which is unknown [117]. Additionally, in ants there exists a large family ‘9-exon’ ORs which have been shown to be specific for CHCs and candidate pheromones [120].

Little is known about the origins of specificity in insect ORs, though a range of functional assays including mutation of specific loci within the OR subunit has been performed. Single amino acid residues often dictate specificity—polymorphism or mutation of one specific residue often results in loss of function or different activity in insect ORs [9,105,121,122,123,124,125,126,127]. This suggests a tight and highly specific binding site, though further investigation is required to determine how this may impact enantiomeric discrimination.

### 5.2. Enantioselective Insect ORs

Due to the low specificity of soluble periphery proteins in insect olfaction (i.e., OBPs or PBPs), it seems logical that ORs comprise the major discriminatory system in insect olfaction. However, as previously stated, not all ORs show high levels of specificity, with very few demonstrating activity with one of the two enantiomers despite the activity of this specificity being observed in electrophysiological and behavioral studies (Table 1). Additionally, few functional studies of insect ORs screen multiple enantiomers of chiral components due to the inaccessibility of non-naturally occurring components.

**Figure 8:**
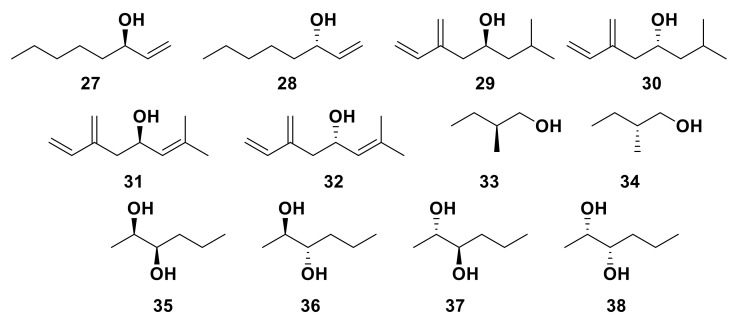
The chemical structures of *A. aegypti* kairomone (*R*)-1-octen-3-ol **27** and (*S*)-1-octen-3-ol **28**, the pheromone components (*S*)-ipsenol **29**, (*R*)-ipsenol **30**, (*R*)-ipsdienol **31** and (*S*)-ipsdienol **32** of the Eurasian spruce bark beetle, *Ips typographus* and the pheromone components (*S*)-2-methyl-1-butanol **33**, (*R*)-2-methyl-1-butanol **34**, (*2R,3R*)-2,3,-hexanediol **35**, (2*R*,3*S*)-2,3-hexanediol **36**, (2*S*,3*R*)-2,3-hexandiol **37** and (2*S*,3*S*)-2,3-hexandiol **38** of the cerambycid beetle *Megacyllene caryae*.

An enantioselective OR has been identified in the Yellow Fever mosquito, *Aedes aegypti* [128]. AaegOR8 co-expressed with the *A. aegypti* ORCO (AaegOR7) was shown to have a significantly higher response to the kairomone (*R*)-1-octen-3-ol **27** than the respective enantiomer, (*S*)-1-octen-3-ol **28** (Figure 8) [128,129], although a small response was observed from (*S*)-1-octen-3-ol **28** and was attributed to a 0.1% enantiomeric impurity [129]. Similar activity has been observed between conserved receptors from other mosquito species (*Toxorhynchites amboinensis*, *Culex quinquefasciatus* and *Anopheles gambiae*) [122,130,131,132]. An OR from *Anopheles gambiae*, AgamOR29, also shows differential responses to the racemate (*R/S*)-linalool and the specific enantiomer (*R*)-linalool **18**, suggesting enantiomeric specificity [45]. Furthermore, two ORs from the Eurasian spruce bark beetle, *Ips typographus*, were shown to possess enantio-discriminatory ability. After initial responses to the racemic mixtures of pheromone components, (*R/S*)-ipsenol (**29** and **30)** and (*R/S*)-ipsdienol (**31** and **32**) (Figure 8), were observed, enantiospecific functional assays were conducted. ItypOR46 was shown to be most responsive to (*S*)-ipsenol **29** and ItypOR49 to (*R*)-ipsdienol **31** [104]. Some ORs have also been shown to respond to multiple enantiomers, though some enantiomers induce slightly higher responses. ORs from the cerambycid beetle, *Megacyllene caryae,* display flexibility when responding to various enantiomers of pheromonal components 2-methyl-1-butanol (**33** and **34**) and 2,3-hexanediol (**35**, **36**, **37** and **38**) [133].

In most cases of enantioselective insect ORs, a racemic mixture of the two components was screened before enantiospecific functional assays were conducted. This further highlights the need for additional exploration where responses are seen from a racemic mixture. Chirality holds biological importance, and it is unlikely two enantiomers of a compound will have an identical activity to one another.

Observed enantiomeric discrimination by insect ORs indicates either a highly specific binding site, or multiple potential sites with only one site activating the receptor. Multiple binding sites have been predicted in insect ORs. Molecular modeling and docking identified two potential binding sites for ligands in ItypOR46 [104]. Other studies of ligand-binding interactions with insect ORs have suggested the existence of multiple binding sites. A study investigating potential allosteric agonist interactions with *A. gambiae* ORCO demonstrated that different ligands may bind simultaneously to different sites [134]. Additionally, mutations in the C-terminal helix (7) of CquiOR8 altered enantioselectivity, indicating the importance of specific loci within the OR unit [122]. Studies with achiral ligands, such as (*Z*)-11-tetradecenyl acetate and Lepidopteran ORs also suggested there may exist multiple ORs for the same compound that recognize different conformations or stereochemistries [135]. Presently, there appear to be few examples of enantiomeric discrimination by ORs, though this may be due to difficulty functionally screening with multiple enantiomers or focus on insect species with no chiral pheromone components.

### 5.3. Structural Studies of Insect ORs

Despite extensive functional studies, the structure of insect OR-ORCO complexes remains elusive. The structure of the ORCO from the fig wasp, *Apocrypta bakeri* was determined as a heteromeric tetramer by Butterwick et al. (2018) using cryo-EM techniques [7]. This has led to large advances in knowledge of the assembly and functionality of ORCO-OR tetramers, including their properties as an ion channel for olfactory signal transduction. Structurally, ORCO can be divided into two domains including four loose peripheral transmembrane domains and a single central anchor domain [7]. Cryo-EM data also confirmed the inverse heptahelical topology [7,9]. Comprehensive structural data can be used to sufficiently model and assess the binding activity of ORs and explain the range of critical residues that have been identified [9,104,124,125,126,127].

Following elucidation of the structure of ORCO from *A. bakeri*, a structure for a homomeric broadly tuned insect OR was elucidated [9]. The structure of OR5 from the jumping bristletail, *Machilidae harabi,* was elucidated as a homomeric OR (MharOR5) activated by a broad range of ecologically relevant olfactory ligands, including the mosquito repellent DEET and eugenol [9]. The ligand binding and channel opening of the OR was resolved, revealing a clear ligand-binding pocket and hydrophobic interactions between the ligands and critical residues. The ligand-binding site was a 15Å-deep pocket within the extracellular portion of the complex, which was shown to be highly conserved across different insect species [9]. Assessment with other ligands also demonstrated this binding pocket to be highly promiscuous, but unable to accommodate all ligands which are known to activate or inhibit the receptor, suggesting the potential for multiple binding sites or additional allosteric sites [9].

**Figure 9:**
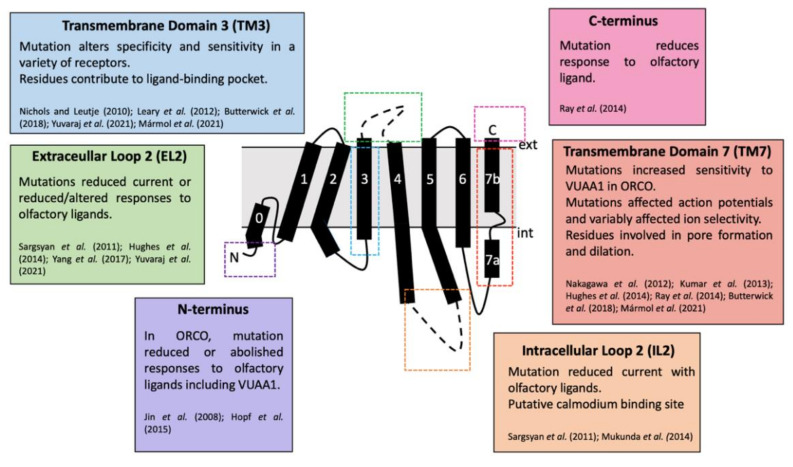
Topology of an ORCO subunit with key loci for activity highlighted [1,7,9,104,124,126,127,136–142]. Not included are transmembrane domains 2,4,5 and 6, extracellular loop 3 and intracellular loop 3, which all possess additional roles in affecting sensitivity to ligands, ion selectivity and response ratios [9,96,123,127,138,143].

The architecture of the odorant-binding pocket of the *M. harabi* OR5 included highly conserved residues that have previously been identified as important in ligand binding [9]. Before a full structure of an insect OR was available, data relied on mutagenesis studies. Maps of critical loci within the OR could be generated by mutating suspected key residues and observing functional changes. Multiple mutagenesis experiments have been performed with ORCO and ORs across a variety of species, many of which aligned with key sections of the final ORCO structure [7]. Mutations in both extracellular loop 2 (EL2) and transmembrane domain 3 (TM3) both generally resulted in responses to olfactory ligands, either altering, reducing or abolishing specificity [105,126,136,137,138]. This functional change can also occur with mutations in the N- and C-termini, transmembrane domains 2,4 and 6 (TM2, TM4 and TM6), extracellular loop 3 (EL3) and intracellular loop 3 (IL3) [123,138,139,140,141,142,143]. Intracellular loop 2 (IL2) is predicted to be a calmodium, a Ca^2+^ regulator protein, binding site, and IL2, transmembrane domains 5–7 (TM6) all affect current and ion selectivity and permeability due to their location around the ORCO-OR complex ion channel pore (Figure 9) [7,96,124,127,138,139,142,144].

Investigations of OR function can utilize computational techniques, specifically modeling and docking analysis, with the limited data that are available [103,104,105,145]. Additional structural data will only improve the accuracy and viability of these techniques [103,104,105,145]. Unfortunately, no structural data exist to date for either a highly specific OR or a heteromeric complex between an OR and ORCO. Progress in understanding how specificity arises in insect ORs, and the subsequent mode of action, will rely on advances in structural biology studies.

## 6. Future Research Directions

### 6.1. Alternative Targets

Enantioselectivity may arise elsewhere in the insect olfactory system. Enantioselective ORNs have been described, such as an ORN from the cabbage moth, *Mamestra brassica,* that is most responsive to (*R*)-linalool **18**, but it is predicted this selectivity arises from the OR expressed within the neuron [44]. Additionally, pheromone-degrading enzymes (PDEs) have demonstrated chiral discrimination in *P. japonica* [146].

Another group of proteins commonly studied in the insect olfactory system is ionotropic receptors (IRs). IRs have many roles in insects, including olfactory perception, in addition to taste, hygroperception (humidity sensing) and cool temperature sensing [147]. IRs, like ORs, are ligand-gated cation channels, though they possess a different general structure. IRs are thought to possess a similar domain topology and overall homology with glutamate receptors [2]. IRs and ORs do not overlap in odor specificity, and IRs mainly respond to amines, aldehydes and acids [147]. IRs have the potential to be enantioselective as shown by selective responses to L-amino acids in *Drosophila* taste perception [148].

### 6.2. Applications of Enantiomeric Discrimination

Many insect species have indirect and direct detrimental effects on human health. Providing a fundamental understanding of insect olfactory systems and enantioselectivity of pheromones and other semiochemicals has the potential to underpin future pest management approaches [3,4,149]. An understanding of how specificity occurs in insect olfaction will shed light on the overall olfactory recognition process. Presently, it is unclear how this specificity arises, and often olfactory proteins are shown to be broadly tuned to a multitude of ligands. Elucidation of differences between highly specific and broadly tuned OBPs and ORs could provide insights into the evolution of specific olfactory responses and discrimination between complex mixtures of compounds. Beyond fundamental understanding, understanding enantiomeric discrimination in insect olfaction may have potential practical applications. These may include manipulation of the insect olfactory system or design of novel olfactory ligands for pest management, and the use of olfactory proteins or modified olfactory proteins in biosensor systems [5,150,151].

## 7. Conclusions

Enantiomeric discrimination plays an important role in insect olfaction and insect chemical ecology. However, how highly specific recognition arises is still unclear. First, the specific role or mode of action of OBPs remains unclear. Though multiple studies explore OBP-ligand binding specificity, it is unclear whether conformational changes, induced by ligand binding and other physiological changes, are a critical component of OBPs function. Investigation into OBP activity needs to consider the screening of multiple enantiomers and not only the racemate or biologically active enantiomer. Furthermore, a more in-depth analysis of the structure and conformational changes induced in OBPs is required. Finally, it appears high specificity only arises in PBPs that specifically bind pheromones. The only example of enantiomeric discrimination by OBPs is by PBPs from *L. dispar*, which show differential responses to the sex pheromone component (*7R,8S)-*(+)-disparlure **2**, and the enantiomer (*7S,8R)*-(-)-disparlure **3** [10,111]. Refinement of OBP classification may be required to easily divide general or non-specific OBPs and highly specific PBPs and their structure, conformation changes and subsequent role.

ORs are a much more convincing target for enantiomeric discrimination. Examples of enantiomeric discrimination by insect Ors exist, indicating these proteins must play an important role in this important process [45,104,122,128,130,133]. Unfortunately, little is understood about the activation of the receptor and opening of the ion channel by a ligand. With such limited structural data available, predictions and explanations of activity are difficult. Mutagenesis studies provide some insight, but additional structural data are needed for progress in understanding these proteins.

Overall, there are very few examples of enantiomeric discrimination by peripheral olfactory proteins in insects. Additional structural and functional data are required for better understanding, and future studies investigating the ligand-binding ability of OBPs and ORs should include both enantiomers of a chiral ligand where possible, especially in the case of highly specific pheromones.

## Figures and Tables

**Table 1:** Insect odorant receptors that possess enantioselectivity experimentally. The suggested active enantiomer is highlighted in bold for each receptor.

Species	Common Name	Receptor	Ligands	Reference
*Aedes aegypti*	Yellow Fever Mosquito	AaegOR8	**(*R*)-1-Octen-3-ol** (*S*)-1-Octen-3-ol	Bohbot and Dickens, 2009 [128]
*Culex quinquefasciatus*	Southern House Mosquito	CquiOR8		Hill et al., 2015 [122]
*Toxorhynchites amboinensis*	Elephant Mosquito	TambOR8		Dekel et al., 2016 [130]
*Anopheles gambiae*	Malaria Vector Mosquito	AgamOR29	(*R*)-Linalool **(*S*)-Linalool**	Huff and Pitts, 2019 [45]
*Megacyllene caryae*	Hickory Borer	McarOR20	**(2S,3R)-2,3-Hexanediol** (2R,3S)-2,3-Hexanediol (2S,3S)-2,3-Hexanediol	Mitchell et al., 2013 [133]
*Ips typographus*	Eurasian spruce Bark Beetle	ItypOR46	(*R*)-Ipsenol ***(*S*)-Ipsenol***	Yuvaraj et al., 2021 [104]
		ItypOR49	**(*R*)-Ipsendiol** (*S*)-Ipsendiol	

## Data Availability

No new data were created or analyzed in this study. Data sharing is not applicable to this article.
